# Characterization of per- and polyfluoroalkyl substances (PFAS) in AFFF-contaminated soil by photocatalytic oxidation (PhotoTOP)

**DOI:** 10.1007/s00216-025-06208-0

**Published:** 2025-11-11

**Authors:** Catharina Capitain, Christian Zwiener

**Affiliations:** Environmental Analytical Chemistry, Department of Geosciences, University of Tübingen, Schnarrenbergstraße 94-96, 72076 Tübingen, Germany

**Keywords:** AFFF, PFAS, Soil, Photocatalytic oxidation, HRMS, NTS

## Abstract

Per- and polyfluoroalkyl substances (PFAS) comprise a diverse group of thousands of compounds, for which only a limited number of analytical reference standards are available. This presents significant challenges for the identification and quantification of PFAS in environmental samples. The PhotoTOP assay addresses this issue by photocatalytic oxidation of PFAS precursors into quantifiable perfluoroalkyl carboxylic acids. In this study, the PhotoTOP was applied for the first time to both aqueous film-forming foam (AFFF)–related PFAS standards and AFFF-contaminated soil. Precursors, intermediates, and final oxidation products were identified using target analysis and non-target screening (NTS). Numerous novel PhotoTOP intermediates were detected, such as perfluoroalkyl sulfonamide–based and fluorotelomer sulfonamide–based compounds, dimers, fluorotelomer betaines, and unsaturated perfluoroalkyl sulfonic acids. Mass balance analysis based on semiquantitative NTS (qNTS) data revealed near-complete conversion of PFAS precursors in both standards and contaminated soil. Additionally, transformation kinetics were investigated, providing insight into the behavior and persistence of PFAS precursors during PhotoTOP treatment and their potential fate in the environment.

**Graphical abstract:**

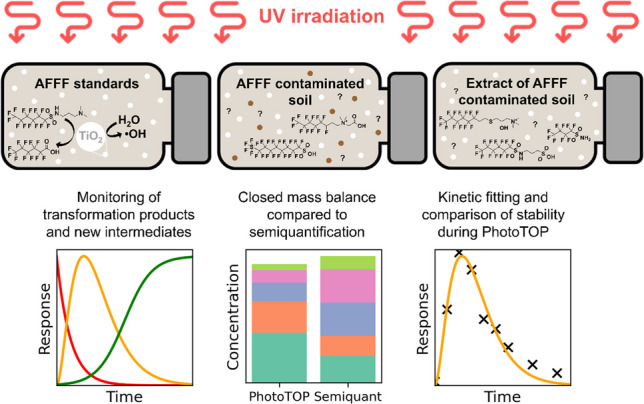

**Supplementary Information:**

The online version contains supplementary material available at 10.1007/s00216-025-06208-0.

## Introduction

Per- and polyfluoroalkyl substances (PFAS) are a large group of synthetic chemicals known for their exceptional stability, resistance to heat, and water- and oil-repellent properties [1]. Due to these characteristics, PFAS have been widely used in various industrial and consumer products, including non-stick cookware, water-resistant fabrics, and food packaging [2, 3]. However, their environmental persistence [4] and potential health risks have raised significant concerns, as PFAS can accumulate in living organisms and are associated with adverse effects on human health, such as endocrine disruption and carcinogenicity [5–8].

One major source of PFAS contamination is aqueous film-forming foams (AFFFs), which are specific firefighting foams used to suppress fuel-based fires [9, 10]. AFFFs contain PFAS to create a stable film that quickly smothers flames and prevents re-ignition [11]. Despite their effectiveness, AFFF use has led to widespread environmental contamination, especially around military bases, airports, and industrial sites where they have been used extensively [9, 10, 12–14]. As a result, understanding and mitigating PFAS contamination from AFFF is a critical focus of environmental research and regulation.

PFAS encompass a highly diverse group of compounds, with over 4700 distinct PFAS identified [15]. However, only a limited number of analytical reference standards is available for these compounds, making their identification and quantification challenging. Semiquantitative non-target screening (qNTS) is necessary to address this limitation, but it requires the use of high-resolution mass spectrometry (HRMS) and comprehensive data analysis workflows, which are labor-intensive and time-consuming [16, 17].

Another approach to estimate the total PFAS load in environmental samples is the total oxidizable precursor (TOP) assay [18]. The TOP assay is a method used to assess PFAS precursor compounds that are difficult to detect directly but can be converted into stable, measurable end products through oxidation. In this assay, PFAS precursors are oxidized by strong oxidizing agents into perfluoroalkyl carboxylic acids (PFCAs) and sulfonic acids (PFSAs) that are easier to analyze. By comparing PFAS concentrations before and after oxidation, the amount of initially undetectable precursor compounds can be estimated [18–22]. The TOP assay provides a more comprehensive understanding of PFAS contamination, as it reveals hidden or precursor-based PFAS that might otherwise go undetected in standard analyses.

The PhotoTOP assay is an alternative to the traditional TOP assay, with a key difference in the oxidation process. While the TOP assay relies on strong chemical oxidizing agents to transform PFAS precursor compounds into stable, measurable end products, the PhotoTOP assay uses ultraviolet (UV) light in combination with the catalyst titanium dioxide (TiO_2_) to achieve transformation through photochemical oxidation [23]. The main advantage of the PhotoTOP compared to the TOP assay is the absence of high salt concentrations, allowing for direct injection of samples without requiring any cleanup. Additionally, the method results in less shortening of the perfluoroalkyl chain length compared to the TOP assay, leading to more meaningful results. The use of UV light and TiO_2_ in the PhotoTOP assay also ensures the continuous generation of oxidants, which ensures complete oxidation of PFAS precursor compounds, and the ability to collect sampling time points during the assay allows for monitoring, improving the accuracy and reliability of the results [24].

The objectives of this study were to conduct the PhotoTOP assay on AFFF standards to identify transformation products (TPs) and pathways, as well as to apply the assay to heavily contaminated soil samples from Reilingen in Southwest Germany. The PhotoTOP assay was applied both directly to the contaminated soil and to soil extract to investigate PFAS extractability and matrix-related effects. The resulting data provide insights into potential TPs and allow for validation of the qNTS data published previously [25], which combined matrix-matched calibration and ionization class-specific average calibration curves. Additionally, this study examines the stability and transformation kinetics of precursors.

## Materials and methods

### Chemicals and reagents

Methanol (MeOH), ammonium acetate (NH_4_Ac), and water were of optima LC-MS grade and were ordered from Thermo Fisher Scientific. Titanium(IV) dioxide (TiO_2_ anatase, powder, 99.8% trace metal basis) was purchased from Sigma-Aldrich. A PFAS standard mixture containing 51 authentic reference standards was used for identification. Detailed information on the reference standards can be found in the Electronic Supplementary Material (ESM; Table S1).

### Site characteristics, soil sampling, and processing

Soil samples were collected from a site in Reilingen in Southwest Germany, where a significant fire incident occurred at a mattress warehouse in 2008. During this event, firefighting efforts involved multiple local fire departments and extensive use of various AFFF formulations, which drained into a nearby irrigation trench (*Nachtwaidgraben*) and impacted an adjacent agricultural field. Soil samples from the *Nachtwaidgraben* were collected and processed as described by Schüßler et al. (2024) [26]. Briefly, in May 2023, drill core sampling was conducted to a depth of 3 m. For this study, only the uppermost soil section (0–0.5 m depth) was analyzed. This sample represents a composite of four drill cores collected in a 1 × 1 m grid. The soil sample was dried at 40 °C, ground, and sieved to a remaining fraction of ≤ 1.6 mm. The water content was 12.6 w/w %.

### Photocatalytic oxidation

The previously developed PhotoTOP protocol [23] was applied to AFFF PFAS reference standards, to the collected soil sample, and to a methanolic extract derived from the soil sample.

AFFF standards (6:2 fluorotelomer sulfonamide propyl betaine (FTSAm-Pr-B; Capstone B), 6:2 fluorotelomer sulfonamide propyl dimethylamine oxide (FTSAm-Pr-DiMeNO; Capstone A), 5:3 fluorotelomer betaine (FTB), 5:1:2 FTB, perfluorohexane sulfonamide (PFHxSAm), perfluorooctane sulfonamide (PFOSAm), perfluorohexane sulfonamide propyl dimethylamine (PFHxSAm-Pr-DiMeAm)) were pipetted into individual 20-mL Pyrex EPA screw-cap glass vials, reaching a final concentration of 2.3 µg. To each vial, 10 mg of TiO_2_ was added.

For soil treatment, 100 mg of soil was weighed into 20-mL Pyrex EPA screw-cap glass vials. Each vial was supplemented with 30 mg TiO_2_ and 200 µL of MeOH to facilitate the coating of the TiO_2_ particles.

For the PhotoTOP of soil extracts, 15 g of soil was extracted in 15 mL of MeOH. The samples were sonicated in an ultrasonic bath for 1 h, shaken on a rotary shaker for 24 h, and centrifuged at 7197 relative centrifugal forces (rcf) for 20 min. After centrifugation, the supernatant was decanted, and 9 mL of the supernatant (corresponding to the extract from 9 g of soil) was pipetted into a 20-mL EPA screw-cap glass vial and evaporated under a gentle stream of nitrogen. Subsequently, 30 mg of TiO_2_ and 200 µL of MeOH were added to the vial for resuspension and coating of the particles.

All sample setups (standards, soil, and soil extract) were thoroughly mixed, sonicated for 1 min to disperse any particle clumps, and left uncovered overnight to allow complete evaporation of MeOH. The following day, 23 mL of water and a magnetic stir bar were added to each vial, and the mixtures were sonicated for 5 min to ensure thorough dispersion.

The vials were then transferred to a UVA Cube 400 (Hönle UV Technology, equipped with a 1200-W lamp; for details, see Zweigle et al. (2022) [23]) and irradiated for 5 h (standards) or 46 h (soil and soil extract samples), respectively. Samples were collected at regular intervals: for standard samples, 100 µL was taken and diluted with 900 µL of MeOH. For soil and soil extract samples, 400 µL was taken and combined with 400 µL of MeOH. These were then centrifuged for 30 min at 20,817 rcf, and 500 µL of the supernatant was transferred into HPLC vials for subsequent analysis.

During the 46-h irradiation process, intervals of irradiation were interrupted for up to several days, and samples were stored at room temperature. After each interruption, the vials were sonicated for 5 min and sampled before resuming irradiation.

### Instrumental analysis

Target PFAS (confidence level 1, Table S1) were quantified with high-performance liquid chromatography (1290 II HPLC from Agilent Technologies, Waldbronn, Germany) coupled to a 6490 triple quadrupole mass spectrometer (Agilent Technologies, Santa Clara, USA; HPLC-QqQ-MS). The separation was conducted on a Waters Acquity BEH C_18_ column (2.1 mm × 100 mm, 1.7 μm particle size) at a flow rate of 0.4 mL/min and maintained at a column temperature of 60 °C. The injection volume was 5 μL for all samples. An 8-min gradient elution was utilized with eluent A consisting of 95% H_2_O and 5% MeOH with 2 mM NH_4_Ac, and eluent B composed of 95% MeOH and 5% H_2_O also with 2 mM NH_4_Ac, starting with 60% eluent B and increased linearly to 100% B over 3.5 min, where it was held constant until 6.0 min. At 6.1 min, the gradient returned to the initial condition of 40% B and was maintained until 8.0 min for re-equilibration before the next injection. Electrospray ionization was operated in negative and positive mode, and every analyte was measured with two mass transitions, except for perfluorobutanoic acid (PFBA) and perfluorooctane sulfonamide (PFOSAm), for which only one product ion was available. In addition to duplicate sample injections, the analysis included a blank, a seven-point calibration curve of the PFAS standard mixture (0.05–10 µg/L, *R*^2^ > 0.99), and quality controls (QCs; PFAS standard mixture, ± 10%) every tenth injection to ensure data accuracy. Details on the limit of detection can be found in Table S2.

For suspect and non-target screening of PFAS identified by Schüßler et al. (2024) [26] (at confidence levels 2 and 3), high-performance liquid chromatography (1290 HPLC from Agilent Technologies, Waldbronn, Germany) coupled to a quadrupole time-of-flight mass spectrometer with an electrospray ionization source (6550 QTOF, Agilent Technologies, Santa Clara, USA; HPLC-ESI-QTOF-MS) was applied. The separation was conducted on an Agilent Poroshell 120 EC-C_18_ column (2.1 mm × 100 mm, 2.7 µm particle size) at a flow rate of 0.3 mL/min and maintained at a column temperature of 40 °C. A sample injection volume of 10 µL was used. A 23-min gradient elution was utilized with identical solvents as for target analysis. The gradient program started with 15% eluent B and gradually increased to 100% over 10 min, then held at 100% B for 5 min. Following each run, an 8-min post-run equilibration returned conditions to 15% B to prepare for subsequent injections. For mass spectrometric detection, ionization was conducted separately in both positive and negative electrospray ionization (ESI+/ESI−) modes to enable the detection of anionic, cationic, and zwitterionic analytes. Data acquisition was performed using the QTOF mass spectrometer in a scan or data-dependent MS/MS (ddMS^2^) mode. A threshold of 1000 counts was set to trigger MS^2^ acquisition, and each precursor m/z was excluded for 0.5 min after collecting three MS^2^ spectra. Both MS^1^ and MS^2^ spectra acquisition rates were set at 3 spectra per second, with a narrow isolation window of 1.3 m/z. Collision energy for MS^2^ was m/z-dependent and calculated using the formula: CE (m/z) = 3 (m/z)/100 + 15 eV, employing collision-induced dissociation. This equation was optimized for PFAS and was found to provide good collision behavior for these compounds. In addition to duplicate sample injections, the analysis included a blank and QCs (± 10%) every fifteenth injection to ensure data accuracy.

Information on the detected PFAS classes and compounds, including acronyms, full names, molecular formulas, mass-to-charge ratios *m*/*z*, ionization polarity, confidence levels, and the oxidized matrices in which each compound was identified, can be found in Table S3 and S4. For improved readability, all PFAS are referred to by their acronyms in the following sections.

### Data analysis

Concentrations were calculated using Agilent MassHunter Quantitative Analysis 10.0 software based on a seven-point calibration curve. The mass balance was calculated by comparing the summed molar concentrations before and after oxidation, or from qNTS data and after oxidation, assuming 1 mol of precursor yields 1 mol of TP (except for dimers).

To assess the kinetics and stability of the different PFAS during the PhotoTOP, the transformation and formation curves were fitted using four different models, depending on the curve shape: logistic formation (Eq. 1), exponential decay (Eq. 2), a combination of logistic formation and exponential decay (Eq. 3), and a two-step exponential formation and decay model (Eq. 4) with concentration *C*(*t*), initial concentration *C*_start_, maximum concentration *C*_max_, time when the formation rate is at its maximum *t*_max_, formation rate *k*_f_, and decay rate *k*_d_:

1 $${\mathrm{C}}\left({\mathrm{t}}\right)={\mathrm{C}}_{\mathrm{start}}+\frac{{\mathrm{C}}_{\mathrm{max}}-{\mathrm{C}}_{\mathrm{start}}}{{1}+{\mathrm{e}}^{-{\mathrm{k}}_{\mathrm{f}}\,\cdot\, ({\mathrm{t}} \, - {\mathrm{t}}_{\mathrm{max}})}}$$

2 $${\mathrm{C}}\left({\mathrm{t}}\right)={\mathrm{C}}_{\mathrm{start}}\,{\cdot\,{\mathrm{e}}}^{-{\mathrm{k}}_{\mathrm{d}}\,\cdot \,{\mathrm{t}}}$$

3 $${\mathrm{C}}\left({\mathrm{t}}\right)={\mathrm{C}}_{\mathrm{start}}\cdot \frac{{\mathrm{C}}_{\mathrm{max}}}{{1}+{\mathrm{e}}^{-{\mathrm{k}}_{\mathrm{f}}\cdot ({\mathrm{t}} \, - {\mathrm{t}}_{\mathrm{max}})}}\,{\cdot\, {\mathrm{e}}}^{-{\mathrm{k}}_{\mathrm{d}}\,\cdot\, {\mathrm{t}}}$$

4 $${\mathrm{C}}\left({\mathrm{t}}\right)={\mathrm{C}}_{\mathrm{start}}{\cdot \,{\mathrm{e}}}^{-{\mathrm{k}}_{\mathrm{d}}\,\cdot\, {\mathrm{t}}}+{\mathrm{C}}_{\mathrm{max}}\,{\cdot\, ({1}-{\mathrm{e}}}^{-{\mathrm{k}}_{\mathrm{f}}\,\cdot\, {\mathrm{t}}})$$

The RMSE (root mean square error) was used to evaluate the goodness of fit between the observed and predicted data. It was first calculated as the square root of the mean squared difference between observed and predicted values. The normalized RMSE was then obtained by dividing the RMSE by the maximum observed value, with lower values indicating better agreement: values below 10% were considered good, 10–20% acceptable, and above 20% suboptimal.

To investigate the influence of perfluorinated chain length on transformation kinetics, Spearman correlations were calculated between chain length and the formation or decay rates within individual PFAS classes. Only PFAS classes with at least two homologs were included in the analysis. Spearman’s *ρ* was computed using Python’s scipy.stats.spearmanr function.

## Results and discussion

### Oxidative conversion of AFFF standards

To evaluate the suitability and performance of the PhotoTOP assay for AFFF oxidation, the transformation of seven known precursors was examined. Therefore, four fluorotelomer PFAS (6:2 FTSAm-Pr-B, 6:2 FTSAm-Pr-DiMeNO, 5:3 FTB, 5:1:2 FTB) and three perfluoroalkane sulfonamide–based PFAS (PFHxSAm, PFOSAm, and PFHxSAm-Pr-DiMeAm) were irradiated, and samples were taken and analyzed.

**Fig. 1:**
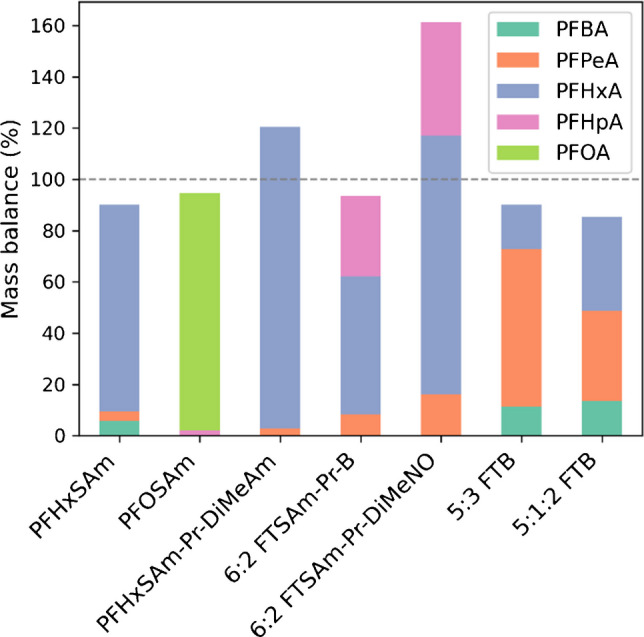
Molar contributions (%) of TPs to mass balance after PhotoTOP of AFFF standards

All precursors and intermediates were transformed within the first hour. Final TPs increased with a time delay, reaching their maximum concentration after no more than 2 h, and remained stable until the end (5 h). After 5 h of oxidation, the mass balance for all substances ranged between 85 and 121% with the exception of 6:2 FTSAm-Pr-DiMeNO, for which the initial concentration was underestimated due to sorption and hence the mass balance was overestimated (Fig. 1, Table S5).

The three perfluoroalkane sulfonamide–based PFAS formed PFCAs, with the length of the perfluorinated carbon chain (n) reduced by 1. Minor fractions of shorter-chain PFCAs were also formed: 11% of the TPs of PFHxSAm and 2% of the TPs of PFOSAm and PFHxSAm-Pr-DiMeAm, respectively (Table S5). However, for PFHxSAm, these probably result largely from impurities in the standard, which already contained the short-chain precursors PFBSAm and PFPeSAm. PFHxSAm-Pr-DiMeAm contained PFHxSAm and 6:2 FTSA prior to irradiation, whereby PFHxSAm might also be a previously formed TP whose concentration further increased during irradiation and was then finally completely transformed.

The four fluorotelomer PFAS formed PFCAs with chain lengths of n (19–43%), n-1 (41–68%), and n-2 (9–16%). 6:2 FTSAm-Pr-B and 6:2 FTSAm-Pr-DiMeNO also formed the intermediates 6:2 FTCA and U-6:2 FTCA. Additionally, 6:2 FTSAm-Pr-DiMeNO contained 6:2 FTSA and 6:2/6:2 FTSAm dimer prior to irradiation, either as impurities or as previously formed transformation products.

The mass balance reached a minimum at 0.5 h for 6:2 FTSAm-Pr-B, both FTBs, and both PFASAms, indicating the presence of potential unknown intermediates. However, with NTS, no further TPs could be identified in this study.

Martin et al. (2019) [27] analyzed selected PFAS standards using the TOP assay, including PFHxSAm, PFOSAm, PFOSAm-Pr-DiMeAm, and 6:2 FTSAm-Pr-B, as in this study. They identified the same TPs for PFHxSAm, PFOSAm, and PFOSAm-Pr-DiMeAm, but observed more pronounced chain shortening for 6:2 FTSAm-Pr-B. Their main TPs were PFBA and PFPeA, whereas the PhotoTOP treatment primarily produced PFHxA and PFHpA, potentially as a result of milder conditions and the absence of sulfate radicals [23].

### Oxidative conversion of contaminated soil

The PhotoTOP was applied to both soil and soil extract for 46 h. The advantage of direct soil oxidation is that it eliminates the need for extraction, thereby avoiding potential analyte losses; however, it also results in a significantly higher matrix load, which can interfere with the oxidation efficiency. In contrast, oxidation of the soil extract allows for the application of larger soil equivalents due to the reduced matrix load. As a result, the soil equivalent in the extract-based approach is approximately 90 times higher compared to the direct soil treatment.

**Fig. 2:**
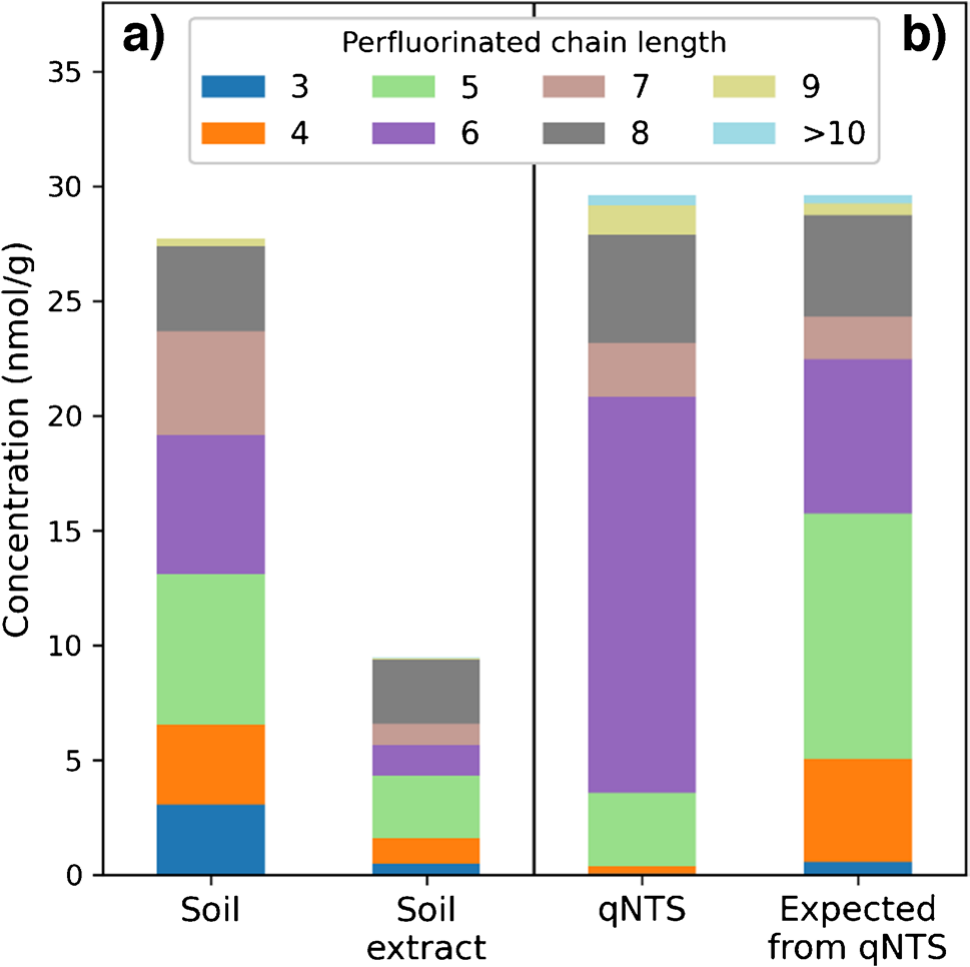
Molar concentrations (nmol/g) of **a**) TPs, stable compounds, and remaining precursors after PhotoTOP with 46-h oxidation of soil and soil extract and of **b**) all PFAS detected by target and qNTS [25], along with expected concentrations based on the observed PhotoTOP transformation patterns

After 46 h of soil oxidation, the summed PFAS concentration, including TPs, stable compounds, and remaining precursors, was 27.6 nmol/g, whereas extract oxidation yielded only 9.5 nmol/g (Fig. 2a, Table S6). This clearly indicates that the extraction process was insufficient to recover the full PFAS burden from the soil. The recently published qNTS approach [25], which referred to the same soil sample, reported a sum of (semi)quantified concentrations of 29.6 nmol/g (Fig. 2b), with 6:2 FTSAm-Pr-B, 6:2 FTSO-(2′)OHPr-TriMeAm, 6:2 FTSy-(2′)OHPr-TriMeAm, PFOS, various FTBs, 6:2 FTSAm, 6:2 and 8:2 FTSA, 6:2 FTSAm-Pr-DiMeAm, and PFHxS, accounting for 90% of the total contamination (Table S7). Only 32.1% of the (semi)quantified PFAS could be recovered through extract oxidation. This poor recovery is due to the different extraction method used in this study, as the qNTS approach employed three sequential extractions with MeOH + 0.4 M ammonium acetate (NH_4_Ac), which is particularly more effective for cationic and zwitterionic substances that are the major contributors to the total PFAS load in this soil. In contrast, 93.2% of the (semi)quantified PFAS could be captured by the direct PhotoTOP assay. This high recovery highlights the effectiveness of the direct oxidation method in capturing a broad range of PFAS of the prevailing contamination, including the fraction missed by extraction.

The molar distribution of perfluorinated carbon chain lengths for all PFAS in the qNTS study [25] was dominated by chains of six (58%) and eight (16%) perfluorinated carbons (Fig. 2b). After PhotoTOP treatment, this distribution of TPs, stable compounds, and remaining precursors became blurred due to chain shortening during the oxidation process, particularly for fluorotelomer PFAS (Fig. 2a), which is consistent with results from AFFF standard oxidation. Applying the observed PhotoTOP transformation pattern from standard oxidation, namely, a perfluorinated chain shortening of n-1 for ECF precursors, and a distribution of 30% n, 55% n-1, and 15% n-2 for fluorotelomer precursors, to the qNTS results (Fig. 2b) shows agreement with the outcomes of the PhotoTOP treatment (Fig. 2a). Specifically, the molar distribution of perfluorinated carbon chain lengths obtained from direct soil oxidation versus those expected from qNTS was 13% vs. 15% (*n* = 4), 24% vs. 36% (*n* = 5), 22% vs. 23% (*n* = 6), 16% vs. 6% (*n* = 7), and 13% vs. 15% (*n* = 8), respectively.

### Transformation kinetics of precursors during photocatalytic oxidation

**Fig. 3:**
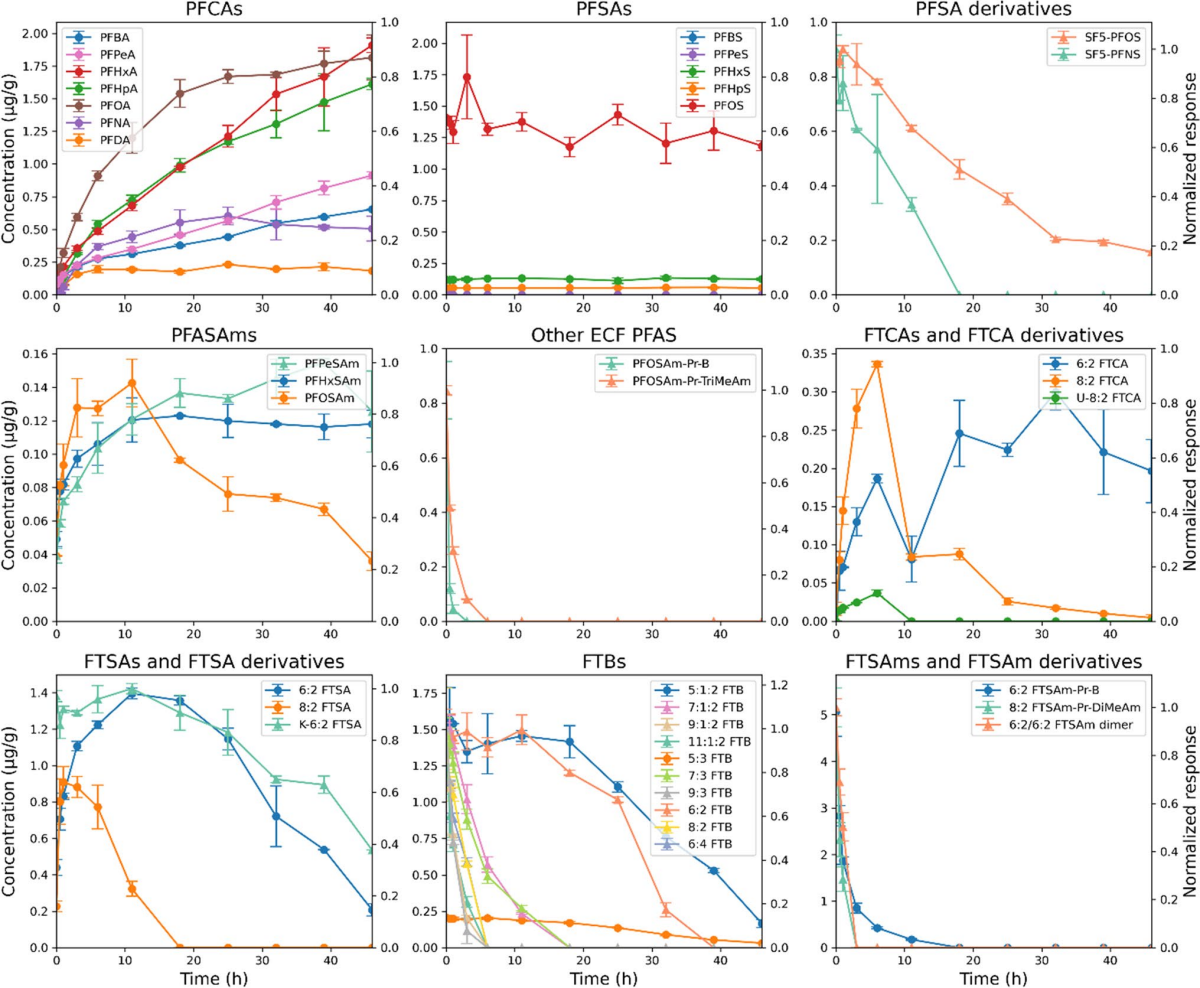
PhotoTOP of contaminated soil containing 124 PFAS. Time trends are shown for precursors, intermediates, and final TPs. Concentrations of level 1 PFAS are plotted on the left axis (μg/g, circles), while normalized responses of level 2 and 3 PFAS are plotted on the right axis (triangles). Error bars represent the standard deviation of duplicate measurements (*n* = 2)

**Fig. 4:**
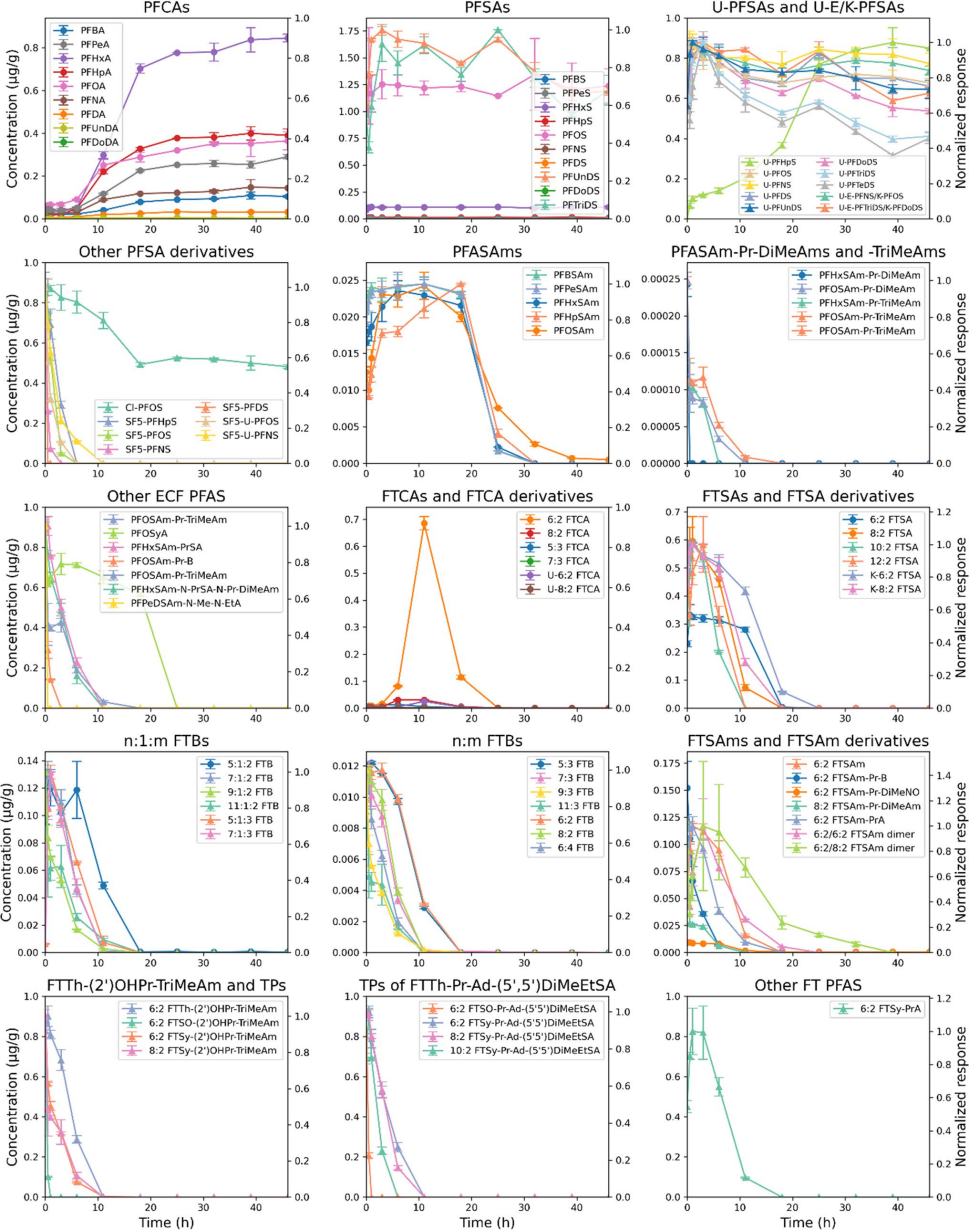
PhotoTOP of extract from contaminated soil containing 124 PFAS. Time trends are shown for precursors, intermediates, and final TPs. Concentrations of level 1 PFAS are plotted on the left axis (μg/g, circles), while normalized responses of level 2 and 3 PFAS are plotted on the right axis (triangles). Error bars represent the standard deviation of duplicate measurements (*n* = 2)

During 46 h of direct soil and soil extract oxidation, precursors transformed, intermediates were formed and subsequently transformed, and PFCAs emerged as main TPs (Figs. 3 and 4).

In the case of direct soil oxidation (Fig. 3), the concentration of some PFCAs, particularly those with perfluorinated chains < 6, was still increasing at the end of the 46-h irradiation period. Additionally, intermediates such as PFASAms, FTCAs, and FTSAs had not yet fully transformed, indicating that photocatalytic oxidation was incomplete. Consequently, the measured total PFAS concentration of 27.6 nmol/g is likely a slight underestimation. Precursors such as SF_5_‑PFSAs, PFOSAm-Pr-B, PFOSAm-Pr-TriMeAm, FTBs, 6:2 FTSAm-Pr-B, 8:2 FTSAm-Pr-DiMeAm, and 6:2/6:2 FTSAm dimer were transformed, with some compounds (e.g., SF_5_-PFOS, 5:1:2 FTB, and 5:3 FTB) being only partially but predominantly converted. To the best of our knowledge, this study is the first to demonstrate the transformation of SF_5_‑PFSAs. Zweigle et al. (2023) [28] reported that SF_5_‑PFSAs were stable in the TOP assay.

As seen from the mass balance, oxidation of soil, rather than soil extract, captures almost all (semi)quantified PFAS. However, the soil oxidation process requires a longer reaction time compared to soil extract oxidation (Figs. 3 and 4), and the higher soil equivalent used in the extract-based approach results in a higher response during measurement and therefore a greater number of detectable PFAS: 42 in the case of direct soil oxidation versus 91 for the extract-based approach (Table S4). In contrast to soil oxidation, by the end of soil extract oxidation (Fig. 4), PFCA concentrations had stabilized and precursors as well as intermediates were nearly completely transformed, indicating that photocatalytic oxidation was likely complete. Due to faster transformation, higher signal responses, and a greater number of detectable PFAS, soil extract oxidation was used to investigate the behavior and fate of different PFAS classes during the PhotoTOP assay. For this purpose, substances were classified as precursors, intermediates, terminal TPs, or stable compounds, based on whether they were formed, showed a rise followed by a decline, underwent transformation only, or remained unchanged (Table S8). However, this classification has some limitations, as some precursors may also act as intermediates, and vice versa. For instance, certain compounds (e.g., Cl-PFOS, K-n:2 FTSA, n:2, n:3, n:1:2 FTB, and n:2 FTSy-Pr-Ad-(5′5′)DiMeEtSA) exhibited a slight initial increase in concentration during the PhotoTOP process. This could be attributed to strong sorption to surfaces such as the glass vial and the soil, followed by desorption during irradiation, possibly triggered by rising temperature and stirring, or to formation as intermediates during the experiment. Moreover, most intermediates were already present prior to irradiation, suggesting they may have formed in the environment through other processes such as biotic and abiotic transformation [29–32]. We have to remember that the contamination already occurred 15 years ago. Consequently, it is likely that many substances are transformed and formed simultaneously during the assay.

Precursors include a variety of perfluorinated PFAS produced by electrochemical fluorination (ECF), such as Cl-PFSA, SF_5_-PFSA, SF_5_-U-PFSA, PFASAm-PrSA, PFASAm-Pr-B, PFASAm-*N*-PrSA-*N*-Pr-DiMeAm, and PFASAm-*N*-Me-*N*-EtA, as well as fluorotelomer-based PFAS such as K-n:2 FTSA, n:1:2, n:2, n:3, and n:4 FTB, n:2 FTSAm-Pr-B, n:2 FTSAm-Pr-DiMeNO, n:2 FTSAm-Pr-DiMeAm, n:2 FTTh-(2′)OHPr-TriMeAm, n:2 FTSO-(2′)OHPr-TriMeAm, FTSy-(2′)OHPr-TriMeAm, n:2 FTSO-Pr-Ad-(5′5′)DiMeEtSA, and FTSy-Pr-Ad-(5′5′)DiMeEtSA. Intermediates comprise ECF-derived compounds including U-PFSA, PFASAm, PFASyA, PFASAm-Pr-DiMeAm, and PFASAm-Pr-TriMeAm, as well as fluorotelomer-derived compounds such as n:2 and n:3 FTCA, U-n:2 FTCA, n:2 FTSA, n:1:3 FTB, n:2 FTSAm, n:2 FTSAm-PrA, n:2/m:2 FTSAm dimer, and n:2 FTSy-PrA. The fate of U-E-PFSA/K-PFSA remains unclear, as it could not be definitively categorized as stable, intermediate, or precursor based on the observed data. PFCAs with perfluorinated chain lengths *n* = 3–11 were formed as terminal TPs, with PFHxA being the predominant TP. In contrast, PFSAs (*n* = 4–13) remained stable throughout the PhotoTOP process, with PFOS reaching the highest concentration. This is the first study to apply PhotoTOP to AFFF-contaminated soil, revealing a wide range of previously uncharacterized precursors and intermediates.

In comparison to the PhotoTOP assay results from previous studies, such as the one by Zweigle et al. (2022) [23], the transformation into PFCAs as main terminal products was comparable. The study also highlighted that the initial PFCA formation was suppressed in the extract with a higher matrix load, as can be clearly seen in this study. However, the extent of transformation and formation delay due to the matrix was less pronounced in Zweigle et al. (2022) [23], likely because the matrix was less concentrated (factor ≈ 8) and the PFAS contamination pattern was simpler (only 8:2 FTSA), which may have affected the overall transformation dynamics.

Martin et al. (2019) [27] also compared the oxidative conversion yields of selected precursors into PFCAs in the TOP assay using both matrix-free (ultra-pure) water and matrix-specific (groundwater) samples. An investigation using 10:2 FTSA as a model compound revealed no significant influence of the groundwater matrix on oxidative conversion. This is likely because the groundwater matrix is less complex than that of soil, consumes fewer hydroxyl radicals, and therefore has a reduced influence on the oxidation process. Both studies also used FTSAs as model compounds, which are characterized by a relatively high susceptibility to oxidative transformation [23, 27].

Al Amin et al. (2023) [21] applied a modified TOP assay to several AFFF formulations and a PFAS-contaminated soil extract. As in this study, the sum of PFCAs increased by several orders of magnitude in both the AFFF samples and the soil extract. Al Amin et al. (2023) [21] used 6:2 and 8:2 FTSAs as oxidation indicators to monitor the progress of oxidation and transformation. However, in this study, these compounds were already transformed after 18 h, whereas other compounds such as PFASAm took much longer to transform and would therefore serve as more reliable indicators. Al Amin et al. (2023) [21] also reported the presence of various perfluorooctane sulfonamide–based PFAS that reached a steady state through the TOP assay, likely because they were simultaneously formed and transformed.

Martin et al. (2019) [27] analyzed AFFF-impacted groundwater samples from an active firefighting training site and reported that a significant portion of the increase in individual PFCAs could be attributed to precursors such as 6:2 FTSAm-Pr-B, FTSAs, PFOSAm-Pr-DiMeAm, and PFHxSAm. Similarly, in this study, 6:2 FTSAm-Pr-B and PFHxSAm were among the compounds with the highest concentrations prior to oxidation.

As noted by Wang et al. (2025) [22], the mass balance after the TOP assay may not be fully closed compared to extractable organofluorine (EOF) results, due to nonoxidizable unknown PFAS, incomplete conversion of unknown PFAS precursors, and the presence of ultra-short-chain PFAS following oxidative conversion. This also applies to the PhotoTOP assay. Therefore, combining oxidative conversion with NTS or EOF analysis, as in this study, is recommended. Additionally, including trifluoroacetic acid (TFA) and HF in the analysis could provide further valuable insights.

To evaluate the kinetics and stability of the different PFAS during the PhotoTOP, the transformation and formation curves were fitted using four different models: logistic formation, exponential decay, a combination of logistic formation and exponential decay, and a two-step exponential formation and decay model. These simplified models were used to describe the overall reaction as a sum of various processes, like transformation of precursors, formation and further transformation of intermediates, and formation of final transformation products. Included are also sorption and desorption on surfaces and TiO_2_ particles, which may delay reactive processes. The models primarily represent the bottleneck reactions that limit the overall reaction rates, but they do not capture all processes involved in detail. Interactions between different PFAS compounds influence the reactions due to competition and transformation among them. As a result, time shifts of the course of reactions can occur, and the models may not always perfectly reflect the system dynamics. The fitted parameters and curves are shown in Table S9 and Figures S1 and S2. The normalized RMSE is below 21%, with a median of 4%, indicating a rather good model fit.

By comparing formation and decay rates, the stability of different PFAS compounds during the PhotoTOP assay can be assessed. Higher decay rates indicate faster transformation processes, which may result from lower chemical stability or stronger sorption affinity to TiO_2_ particles, where OH radicals are generated. The newly identified intermediates, U-PFSAs, exhibit very low decay rates (> 0.7 h^−1^), suggesting high stability under PhotoTOP conditions. PFHxSAm, the perfluorinated analogue of 6:2 FTSAm, shows a higher decay rate than 6:2 FTSAm (0.49 h^−1^ vs. 0.17 h^−1^), indicating it undergoes less simultaneous formation, likely due to a higher abundance of fluorotelomer-based precursors compared to perfluorinated ones. This is supported by the higher formation rate of 6:2 FTSAm (2.08 h^−1^) compared to PFHxSAm (0.51 h^−1^). A comparison between PFASAm-Pr-DiMeAm and PFASAm-Pr-TriMeAm, with perfluorinated chain lengths of *n* = 6 and *n* = 8, shows that PFASAm-Pr-DiMeAm has decay rates approximately seven times higher than PFASAm-Pr-TriMeAm for *n* = 6, and about three times higher for *n* = 8. This indicates that PFASAm-Pr-DiMeAm transforms faster than its trimethylated analogue, likely due to the presence of a free electron pair which can be oxidized. Furthermore, the decay rates of 6:2 FTSO-(2′)OHPr-TriMeAm (2.19 h^−1^ for soil and 4.42 h^−1^ for soil extract) and 6:2 FTSO-Pr-Ad-(5′5′)DiMeEtSA (3.16 h^−1^ for soil extract) are significantly higher than those of 6:2 FTSy-(2′)OHPr-TriMeAm (0.26 h^−1^ for soil and 0.43 h^−1^ for soil extract) and 6:2 FTSy-Pr-Ad-(5′5′)DiMeEtSA (0.23 h^−1^ for soil extract), which in turn are higher than the decay rate of 6:2 FTTh-(2′)OHPr-TriMeAm (0.16 h^−1^ for soil and 0.18 h^−1^ for soil extract). This trend highlights the strong effect of easily oxidizable chemical structure moieties on the overall transformation behavior during the PhotoTOP assay. While the stability observed in the PhotoTOP assay can provide insights into environmental stability, it may not directly reflect real-world behavior, as other processes and environmental conditions can significantly affect PFAS transformation. For example, n:2 FTTh-(2′)OHPr-TriMeAm serves as the precursor for n:2 FTSO-(2′)OHPr-TriMeAm, which is subsequently transformed into n:2 FTSy-(2′)OHPr-TriMeAm. qNTS revealed higher concentrations of 6:2 FTSO-(2′)OHPr-TriMeAm and 6:2 FTSy-(2′)OHPr-TriMeAm compared to their precursor [25]. Therefore, under environmental conditions, the decay of n:2 FTSO-(2′)OHPr-TriMeAm and n:2 FTSy-(2′)OHPr-TriMeAm cannot exceed that of n:2 FTTh-(2′)OHPr-TriMeAm.

Fang et al. (2024) [33] demonstrated in biotransformation experiments in aerobic sludge that 6:2 FTSAm-Pr-B is significantly more stable than 6:2 FTSAm-Pr-DiMeAm and more stable than 6:2 FTSAm-Pr-DiMeNO, which is consistent with the observed stability of their perfluorinated analogues in aerobic soil [34]. In contrast, the results presented here show the opposite trend: 6:2 FTSAm-Pr-B transforms faster than 6:2 FTSAm-Pr-DiMeNO, and PFOSAm-Pr-B transforms faster than PFOSAm-Pr-DiMeAm. Although FTBs have previously been reported to be highly resistant to microbial degradation [35], in this study, under OH radical-mediated conditions, they transformed at rates comparable to those of other precursors.

Zweigle et al. (2023) [28] reported that Cl-PFOS, SF_5_-PFSAs, SF_5_-U-PFSAs, U-PFSAs, and U-E-PFSAs were stable in the TOP assay, whereas, in the PhotoTOP assay, these substances were observed to transform, albeit very slowly. Notably, U-PFSAs, especially U-PFHpS, were even formed as intermediates. This discrepancy may be due to the fact that the TOP assay does not allow for time-resolved monitoring, and therefore transformation processes may not have reached completion.

Spearman correlations between perfluorinated chain length and formation/decay rates indicate a strong positive correlation for soil oxidation, but both strong positive and strong negative correlations for soil extract oxidation (Figure S3). The strong correlation observed in soil oxidation likely reflects that longer homologues have a higher sorption affinity for TiO_2_ particles, leading to faster transformation compared to shorter homologues. In contrast, the strong positive and strong negative correlations in soil extract oxidation may result from strong initial sorption of PFAS to the glass vials. In this case, PFAS must first desorb from the glass before they can sorb to the TiO_2_ particles, disrupting the expected correlation pattern.

This correlation in the PhotoTOP contradicts the observed microbial stability. As Dong et al. (2024) [31] suggested, a longer perfluorinated carbon chain may lead to increased microbial stability. This further highlights that PhotoTOP results may not directly reflect real-world environmental behavior. Nevertheless, it provides valuable insight into which substances are highly stable and which intermediates and TPs can be expected.

## Conclusion

Precursors represent a complex source of persistent perfluoroalkyl acids (PFAAs) in the environment, with many transformation pathways still insufficiently understood. The PhotoTOP approach offers a valuable tool for addressing these knowledge gaps by enabling long-term prediction of TPs and providing insights into precursor fate. Key advantages of the PhotoTOP are its ability to largely preserve the original chain length distribution, resulting in a relatively narrow chain length distribution of the TPs as a mix of n, n-1, and n-2, offering additional information about precursor chain lengths, and the temporal monitoring of PFAS transformation. When combined with NTS, it facilitates comprehensive monitoring of transformation kinetics from precursors through intermediates to final products. Moreover, PhotoTOP serves as a complementary method for validating qNTS. Its application to both soil and soil extracts presents distinct benefits: direct oxidation of soil circumvents limitations due to incomplete extraction, while extract oxidation yields higher PFAS concentrations in measured samples and enhanced analytical sensitivity due to reduced matrix loads. Altogether, the PhotoTOP represents a robust and versatile tool for improving our understanding and assessment of PFAS contamination and transformation in environmental matrices.

## Supplementary Information

Below is the link to the electronic supplementary material.

Supplementary Material 1 (PDF 1.50 MB)

## Data Availability

Data will be made available on request.
