# Supplementary material for: Characterization of per- and polyfluoroalkyl substances (PFAS) in AFFF-contaminated soil by photocatalytic oxidation (PhotoTOP)
Source: Anal Bioanal Chem. 2025 Nov 11;418(12):3555–65. doi: 10.1007/s00216-025-06208-0 (PMC13221326; doi:10.1007/s00216-025-06208-0)
Supplement: Supplementary file 1 — Supplementary Material 1 (PDF 1.50 MB) [file 216_2025_6208_MOESM1_ESM.pdf]

Analytical and Bioanalytical Chemistry

Electronic Supplementary Material

Characterization of per and polyfluoroalkyl substances (PFAS) in AFFF contaminated soil by photocatalytic oxidation (PhotoTOP)

Catharina Capitain[1], Christian Zwiener[1]

1 Environmental Analytical Chemistry, Department of Geosciences, University of Tübingen, Schnarrenbergstraße 94-96, 72076 Tübingen, Germany

Table of content

ESM A Chemicals 3

ESM B Limit of detection 6

ESM C Information on all identified classes/compounds 7

ESM D Results of AFFF standard oxidation 12

ESM E Results after direct soil and soil extract oxidation 13

ESM F Results from qNTS study 14

ESM G Fate of PFAS classes in PhotoTOP 15

ESM H Kinetics 16

ESM I Spearman correlations 25

References 26

ESM A Chemicals

Table S1: Overview about all PFAS reference standards that were included in the PFAS standard mixture and where they were purchased. PFAS originated either from Wellington Laboratories, Guelph, Ontario, Canada (1), Toronto Research Chemicals, North York, Ontario, Canada (2), Dr. Ehrenstorfer, Augsburg, Bavaria, Germany (3) or were custom-synthesized in-house (4).

Acronym	Used standard / purchased chemical	Origin
**PFCAs**		
PFBA	Perfluorobutanoic acid / perfluoro-n-butanoic acid	(1)
PFPeA	Perfluoropentanoic acid / perfluoro-n-pentanoic acid	(1)
PFHxA	Perfluorohexanoic acid / perfluoro-n-hexanoic acid	(1)
PFHpA	Perfluoroheptanoic acid / perfluoro-n-heptanoic acid	(1)
PFOA	Perfluorooctanoic acid / perfluoro-n-octanoic acid	(1)
PFNA	Perfluorononanic acid / perfluoro-n-nonanic acid	(1)
PFDA	Perfluorodecanoic acid / perfluoro-n-decanoic acid	(1)
PFUnDA	Perfluoroundecanoic acid / perfluoro-n-undecanoic acid	(1)
PFDoDA	Perfluorododecanoic acid / perfluoro-n-dodecanoic acid	(1)
PFTriDA	Perfluorotridecanoic acid / perfluoro-n-tridecanoic acid	(1)
PFTeDA	Perfluorotetradecanoic acid / perfluoro-n-tetradecanoic acid	(1)
PFHxDA	Perfluorohexadecanoic acid / perfluoro-n-hexadecanoic acid	(1)
PFODA	Perfluorooctadecanoic acid / perfluoro-n-octadecanoic acid	(1)
**PFSAs**		
PFBS	Perfluorobutanesulfonic acid / potassium perfluoro-1-butanesulfonate	(1)
PFPeS	Perfluoropentanesulfonic acid / sodium perfluoro-1-pentanesulfonate	(1)
PFHxS	Perfluorohexanesulfonic acid / sodium perfluoro-1-hexanesulfonate	(1)
PFHpS	Perfluoroheptanesulfonic acid / sodium perfluoro-1-heptanesulfonate	(1)
PFOS	Perfluorooctanesulfonic acid / sodium perfluoro-1-octanesulfonate	(1)
PFNS	Perfluorononanesulfonic acid / sodium perfluoro-1-nonanesulfonate	(1)
PFDS	Perfluorodecanesulfonic acid / sodium perfluoro-1-decanesulfonate	(1)
PFDoDS	Perfluorododecanesulfonic acid / sodium perfluoro-1-dodecanesulfonate	(1)
**PFPAs**		
PFOPA	Perfluorooctylphosphonic acid	(1)
PFDPA	Perfluorodecylphosphonic acid	(1)
**PAPs**		
6:2/6:2 diPAP	6:2/6:2 phosphoric acid diester / Bis[2-(perfluorohexyl)ethyl] phosphate	(2)
8:2/8:2 diPAP	6:2/6:2 polyfluoroalkyl phosphoric acid diester / Sodium bis (1H, 1H, 2H, 2H-perfluorodecyl) phosphate	(1)
6:2 PAP	6:2 polyfluoroalkyl phosphoric ester / Mono[2-(perfluorohexyl)ethyl] phosphate	(2)

8:2 PAP	6:2 polyfluoroalkyl phosphoric ester / Sodium 1H, 1H, 2H, 2H-perfluorodecyl phosphate	(1)
**PFPiAs**		
C6/C6 PFPiA	C6/C6 Perfluoroalkyl phosphinic acid	(2)
**PASF-based PFAS**		
PFHxSAm	Perfluorohexane sulfonamide	(3)
PFOSAm	Perfluorooctane sulfonamide	(1)
PFOSAm-*N*-Et-*N*-EtA	Perfluorooctane sulfonamide *N*-ethyl *N*-ethanoic acid	(1)
SamPAP	Perfluorooctane sulfonamide ethanol-based phosphate diester /Sodium-2-(N-ethylperfluorooctane-1-sulfonamido) ethyl phosphate	(1)
diSAmPAP	Perfluorooctane sulfonamide ethanol-based phosphate diester/ Sodium bis[2-(N-ethylperfluorooctane-1-sulfonamido) ethyl] phosphate	(1)
**FTCAs**		
6:2 FTCA	6:2 fluorotelomer carboxylic acid / 2-Perfluorohexyl ethanoic acid (6:2)	(1)
8:2 FTCA	8:2 fluorotelomer carboxylic acid / 2-Perfluorooctyl ethanoic acid (6:2)	(1)
5:3 FTCA	5:3 fluorotelomer carboxylic acid/ 3-Perfluoropentyl propanoic acid	(1)
7:3 FTCA	7:3 fluorotelomer carboxylic acid/ 3-Perfluoroheptyl propanoic acid	(1)
**U-FTCAs**		
U-6:2 FTCA	6:2 fluorotelomer unsaturated carboxylic acid/ 2H-Perfluoro-2-octenoic acid (6:2)	(1)
U-8:2 FTCA	8:2 fluorotelomer unsaturated carboxylic acid/ 2H-Perfluoro-2-decenoic acid (8:2)	(1)
**FTSAs**		
6:2 FTSA	6:2 fluorotelomer sulfonic acid / Sodium 1H, 1H,2H,2H-perfluorooctanesulfonate	(1)
8:2 FTSA	8:2 fluorotelomer sulfonic acid / Sodium 1H, 1H,2H,2H-perfluorodecanesulfonate	(1)
**FTMAPs**		
6:2 FTMAP	6:2 Fluorotelomer mercapto alkyl phosphate	(4)
**PFECAs**		
HFPO-Da	2,3,3,3-Tetrafluoro-2-(1,1,2,2,3,3,3-heptafluoropropoxy) propanoic acid	(1)
ADONA	Sodium dodecafluoro-3H-4,8-dioxanonanoate	(1)
**PFESAs**		
9Cl-PF3ONS	Potassium 9-chlorhexadecafluoro-3-oxanonane-1-sulfonate	(1)
11Cl-PF3OUdS	Potassium 11-chloroeicosafluoro-3-oxaundecane-1-sulfonate	(1)
**AFFF-substances**		
6:2 FTSAm-Pr-DiMeNO	6:2 fluorotelomer sulfonamide propyl methylamineoxide / Capstone product A	(3)
6:2 FTSAm-Pr-B	6:2 fluorotelomer sulfonamide propyl betaine / Capstone product B	(3)
PFHxSAm-Pr-DiMeAm	N-[3(dimethylamino)propyl] perfluoro-1-hexanesulfonamide	(3)

5:3 FTB	5:3 Fluorotelomer betaine / 2-[4,4,5,5,6,6,7,7,8,8,8-Undecafluorooctyl) dimethylammonio] acetate	(1)
5:1:2 FTB	5:1:2 Fluorotelomer etaine / 2-[(3,4,4,5,5,6,6,7,7,8,8,8-Dodecafluorooctyl) dimethylammonio] acetate	(1)

ESM B Limit of detection

Table S2: Limits of detection (LOD) for PFAS detected in soil and soil extracts.

Name	LOD for standard oxidation (nM)	LOD in soil (pmol/g)	LOD in soil extract (pmol/g)
PFBA	0.23	108.0	1.2
PFPeA	0.38	174.9	1.9
PFHxA	0.16	73.5	0.8
PFHpA	0.28	126.7	1.4
PFOA	0.12	55.7	0.6
PFNA	0.11	49.7	0.6
PFDA	0.10	44.8	0.5
PFUnDA	0.09	40.9	0.5
PFDoDA	0.08	37.5	0.4
PFBS	0.17	76.9	0.9
PFPeS	0.14	65.9	0.7
PFHxS	0.13	57.7	0.6
PFHpS	0.11	51.2	0.6
PFOS	0.10	46.1	0.5
PFNS	0.09	41.9	0.5
PFDS	0.08	38.4	0.4
PFDoDS	0.07	32.9	0.4
PFHxSAm	0.13	57.8	0.6
PFOSAm	0.10	46.2	0.5
PFHxSAm-Pr-DiMeAm	0.10	47.4	0.5
6:2 FTCA	0.27	122.0	1.4
8:2 FTCA	0.10	48.2	0.5
5:3 FTCA	0.15	67.4	0.7
U-6:2 FTCA	0.14	64.4	0.7
U-8:2 FTCA	0.11	50.3	0.6
6:2 FTSA	0.12	53.9	0.6
8:2 FTSA	0.19	87.3	1.0
5:1:2 FTB	0.12	53.2	0.6
5:3 FTB	0.12	55.5	0.6
6:2 FTSAm-Pr-B	0.18	80.5	0.9
6:2 FTSAm-Pr-DiMeNO	0.09	43.5	0.5

ESM C Information on all identified classes/compounds

Table S3: All detected classes with acronyms and full names.

**Acronym**	**Class**
Cl-PFSA	Chloro substituted perfluoro sulfonic acid
K-n:2 FTSA	Ketone-n:2 fluorotelomer sulfonic acid
n:1:2 FTB	n:1:2 fluorotelomer betaine
n:1:3 FTB	n:1:3 fluorotelomer betaine
n:2 FTB	n:2 fluorotelomer betaine
n:2 FTCA	n:2 fluorotelomer carboxylic acid
n:2 FTSA	n:2 fluorotelomer sulfonic acid
n:2 FTSAm	n:2 fluorotelomer sulfonamide
n:2 FTSAm-PrA	n:2 fluorotelomer sulfonamide propanoic acid
n:2 FTSAm-Pr-B	n:2 fluorotelomer sulfonamide propyl betaine
n:2 FTSAm-Pr-DiMeAm	n:2 fluorotelomer sulfonamide propyl dimethylamine
n:2 FTSAm-Pr-DiMeNO	n:2 fluorotelomer sulfonamide propyl dimethylamineoxide
n:2 FTSO-(2')OHPr-TriMeAm	n:2 fluorotelomer sulfoxide (2')-propanol trimethylamine
n:2 FTSO-Pr-Ad-(5',5')DiMeEtSA	n:2 fluorotelomer sulfoxide propyl amide (5'5')-dimethylethyl sulfonic acid
n:2 FTSy-(2')OHPr-TriMeAm	n:2 fluorotelomer sulfone (2')-propanol trimethylamine
n:2 FTSy-PrA	n:2 fluorotelomer sulfone propanoic acid
n:2 FTSy-Pr-Ad-(5',5')DiMeEtSA	n:2 fluorotelomer sulfone propyl amide (5'5')-dimethylethyl sulfonic acid
n:2 FTTh-(2')OHPr-TriMeAm	n:2 fluorotelomer thio (2')-propanol trimethylamine
n:2/m:2 FTSAm dimer	n:2/m:2 fluorotelomer sulfonamide dimer
n:3 FTB	n:3 fluorotelomer betaine
n:3 FTCA	n:3 fluorotelomer carboxylic acid
n:4 FTB	n:4 fluorotelomer betaine
PFASAm	Perfluoroalkane sulfonamide
PFASAm-*N*-Me-*N*-EtA	Perfluoroalkane sulfonamide *N*-methyl *N*-ethanoic acid
PFASAm-*N*-PrSA-*N*-Pr-DiMeAm	Perfluoroalkane sulfonamide *N*-propyl sulfonic acid *N*-propyl dimethylamine
PFASAm-Pr-B	Perfluoroalkane sulfonamide propyl betaine
PFASAm-Pr-DiMeAm	Perfluoroalkane sulfonamide propyl dimethylamine
PFASAm-PrSA	Perfluoroalkane sulfonamide propyl sulfonic acid
PFASAm-Pr-TriMeAm	Perfluoroalkane sulfonamide propyl trimethylamine
PFASyA	Perfluoro sulfinic acid
PFCA	Perfluoro carboxylic acid
PFSA	Perfluoro sulfonic acid
SF_5-PFSA	Pentafluorosulfanyl perfluoro sulfonic acid
SF_5-U-PFSA	Pentafluorosulfanyl unsaturated perfluoro sulfonic acid

U-E-PFSA/K-PFSA	Unsaturated ether perfluoro sulfonic acid/Ketone perfluoro sulfonic acid
U-n:2 FTSA	Unsaturated n:2 fluorotelomer sulfonic acid
U-PFSA	Unsaturated perfluoro sulfonic acid

Table S4: All detected compounds with formula, mass to charge ratio *m/z*, polarity, confidence level, and the oxidized matrix in which each compound was identified.

**Name**	**Formula**	**m/z**	**Polarity**	**Confidence level**	**Oxidized matrix**
10:2 FTSA	C12 H5 F21 O3 S	626.9542	Negative	Level 2&3	Soil extract
10:2 FTSy-Pr-Ad-(5'5')DiMeEtSA	C19 H18 F21 N O6 S2	818.0145	Negative	Level 2&3	Soil extract
11:1:2 FTB	C18 H13 F24 N O2	732.0617	Positive	Level 2&3	Soil and soil extract
11:3 FTB	C18 H14 F23 N O2	714.0722	Positive	Level 2&3	Soil extract
12:2 FTSA	C14 H5 F25 O3 S	726.9482	Negative	Level 2&3	Soil extract
5:1:2 FTB	C12 H13 F12 N O2	432.0825	Positive	Level 1	Soil and soil extract
5:1:3 FTB	C13 H15 F12 N O2	446.0981	Positive	Level 2&3	Soil extract
5:3 FTB	C12 H14 F11 N O2	414.0916	Positive	Level 1	Soil and soil extract
5:3 FTCA	C8 H5 F11 O2	341.0029	Negative	Level 1	Soil extract
6:2 FTB	C12 H12 F13 N O2	450.0726	Positive	Level 2&3	Soil and soil extract
6:2 FTCA	C8 H3 F13 O2	376.9847	Negative	Level 1	Soil and soil extract
6:2 FTSA	C8 H5 F13 O3 S	426.9676	Negative	Level 1	Soil and soil extract
6:2 FTSAm	C8 H6 F13 N O2 S	425.9837	Negative	Level 2&3	Soil extract
6:2 FTSAm-PrA	C11 H10 F13 N O4 S	498.0038	Negative	Level 2&3	Soil extract
6:2 FTSAm-Pr-B	C15 H19 F13 N2 O4 S	571.0929	Positive	Level 1	Soil and soil extract
6:2 FTSAm-Pr-DiMeNO	C13 H17 F13 N2 O3 S	529.0837	Positive	Level 1	Soil extract
6:2 FTSO-(2')OHPr-TriMeAm	C14 H18 F13 N O2 S	512.0921	Positive	Level 2&3	Soil and soil extract
6:2 FTSO-Pr-Ad-(5'5')DiMeEtSA	C15 H18 F13 N O5 S2	602.0325	Negative	Level 2&3	Soil extract
6:2 FTSy-(2')OHPr-TriMeAm	C14 H18 F13 N O3 S	528.087	Positive	Level 2&3	Soil and soil extract
6:2 FTSy-PrA	C11 H9 F13 O4 S	482.9926	Negative	Level 2&3	Soil extract
6:2 FTSy-Pr-Ad-(5'5')DiMeEtSA	C15 H18 F13 N O6 S2	618.028	Negative	Level 2&3	Soil extract
6:2 FTTh-(2')OHPr-TriMeAm	C14 H18 F13 N O S	496.0955	Positive	Level 2&3	Soil and soil extract
6:2/6:2 FTSAm dimer	C16 H9 F26 N O4 S2	835.9468	Negative	Level 2&3	Soil and soil extract
6:2/8:2 FTSAm dimer	C18 H9 F30 N O4 S2	935.9405	Negative	Level 2&3	Soil extract

6:4 FTB	C14 H16 F13 N O2	478.1039	Positive	Level 2&3	Soil and soil extract
7:1:2 FTB	C14 H13 F16 N O2	532.076	Positive	Level 2&3	Soil and soil extract
7:1:3 FTB	C15 H15 F16 N O2	546.0917	Positive	Level 2&3	Soil extract
7:3 FTB	C14 H14 F15 N O2	514.0854	Positive	Level 2&3	Soil and soil extract
7:3 FTCA	C10 H5 F15 O2	440.9958	Negative	Level 1	Soil extract
8:2 FTB	C14 H12 F17 N O2	550.0662	Positive	Level 2&3	Soil and soil extract
8:2 FTCA	C10 H3 F17 O2	476.9783	Negative	Level 1	Soil and soil extract
8:2 FTSA	C10 H5 F17 O3 S	526.9608	Negative	Level 1	Soil and soil extract
8:2 FTSAm-Pr-DiMeAm	C15 H17 F17 N2 O2 S	613.0825	Positive	Level 2&3	Soil and soil extract
8:2 FTSy-(2')OHPr-TriMeAm	C16 H18 F17 N O3 S	628.08	Positive	Level 2&3	Soil and soil extract
8:2 FTSy-Pr-Ad-(5'5')DiMeEtSA	C17 H18 F17 N O6 S2	718.0217	Negative	Level 2&3	Soil extract
9:1:2 FTB	C16 H13 F20 N O2	632.0693	Positive	Level 2&3	Soil and soil extract
9:3 FTB	C16 H14 F19 N O2	614.0785	Positive	Level 2&3	Soil and soil extract
Cl-PFOS	C8 H Cl F16 O3 S	514.9016	Negative	Level 2&3	Soil extract
K-6:2 FTSA	C8 H3 F13 O4 S	440.9464	Negative	Level 2&3	Soil and soil extract
K-8:2 FTSA	C10 H3 F17 O4 S	540.9401	Negative	Level 2&3	Soil extract
PFBA	C4 H F7 O2	212.9791	Negative	Level 1	Soil and soil extract
PFBS	C4 H F9 O3 S	298.9427	Negative	Level 1	Soil and soil extract
PFBSAm	C4 H2 F9 N O2 S	297.9589	Negative	Level 2&3	Soil extract
PFDA	C10 H F19 O2	512.9601	Negative	Level 1	Soil and soil extract
PFDoDA	C12 H F23 O2	612.9551	Negative	Level 1	Soil extract
PFDoDS	C12 H F25 O3 S	698.9158	Negative	Level 1	Soil extract
PFDS	C10 H F21 O3 S	598.9226	Negative	Level 1	Soil extract
PFHpA	C7 H F13 O2	362.9713	Negative	Level 1	Soil and soil extract
PFHpS	C7 H F15 O3 S	448.9329	Negative	Level 1	Soil and soil extract
PFHpSAm	C7 H2 F15 N O2 S	447.9486	Negative	Level 2&3	Soil extract
PFHxA	C6 H F11 O2	312.9738	Negative	Level 1	Soil and soil extract
PFHxS	C6 H F13 O3 S	398.9362	Negative	Level 1	Soil and soil extract
PFHxSAm	C6 H2 F13 N O2 S	397.952	Negative	Level 1	Soil and soil extract

PFHxSAm-N-PrSA-N-Pr-DiMeAm	C14 H19 F13 N2 O5 S2	607.0592	Positive	Level 2&3	Soil extract
PFHxSAm-Pr-DiMeAm	C11 H13 F13 N2 O2 S	485.0559	Positive	Level 1	Soil extract
PFHxSAm-PrSA	C9 H8 F13 N O5 S2	519.955	Negative	Level 2&3	Soil extract
PFHxSAm-Pr-TriMeAm	C12 H15 F13 N2 O2 S	499.0711	Positive	Level 2&3	Soil extract
PFNA	C9 H F17 O2	462.9626	Negative	Level 1	Soil and soil extract
PFNS	C9 H F19 O3 S	548.9265	Negative	Level 1	Soil extract
PFOA	C8 H F15 O2	412.9659	Negative	Level 1	Soil and soil extract
PFOS	C8 H F17 O3 S	498.9299	Negative	Level 1	Soil and soil extract
PFOSAm	C8 H2 F17 N O2 S	497.9451	Negative	Level 1	Soil and soil extract
PFOSAm-Pr-B	C15 H15 F17 N2 O4 S	643.0551	Positive	Level 2&3	Soil and soil extract
PFOSAm-Pr-DiMeAm	C13 H13 F17 N2 O2 S	583.0332	Negative	Level 2&3	Soil extract
PFOSAm-Pr-TriMeAm	C14 H15 F17 N2 O2 S	599.0648	Positive	Level 2&3	Soil and soil extract
PFOSyA	C8 H F17 O2 S	482.9342	Negative	Level 2&3	Soil extract
PFPeA	C5 H F9 O2	262.9772	Negative	Level 1	Soil and soil extract
PFPeDSAm-N-Me-N-EtA	C18 H6 F31 N O4 S	919.9454	Negative	Level 2&3	Soil extract
PFPeS	C5 H F11 O3 S	348.9394	Negative	Level 1	Soil and soil extract
PFPeSAm	C5 H2 F11 N O2 S	347.9556	Negative	Level 2&3	Soil and soil extract
PFTriDS	C13 H F27 O3 S	748.9127	Negative	Level 2&3	Soil extract
PFUnDA	C11 H F21 O2	562.9562	Negative	Level 1	Soil extract
PFUnDS	C11 H F23 O3 S	648.9194	Negative	Level 2&3	Soil extract
SF_5-PFDS	C10 H F25 O3 S2	706.888	Negative	Level 2&3	Soil extract
SF_5-PFHpS	C7 H F19 O3 S2	556.8978	Negative	Level 2&3	Soil extract
SF_5-PFNS	C9 H F23 O3 S2	656.8902	Negative	Level 2&3	Soil and soil extract
SF_5-PFOS	C8 H F21 O3 S2	606.8943	Negative	Level 2&3	Soil and soil extract
SF_5-U-PFNS	C9 H F21 O3 S2	618.8944	Negative	Level 2&3	Soil extract
SF_5-U-PFOS	C8 H F19 O3 S2	568.8982	Negative	Level 2&3	Soil extract
U-6:2 FTCA	C8 H2 F12 O2	356.9791	Negative	Level 1	Soil extract
U-8:2 FTCA	C10 H2 F16 O2	456.9749	Negative	Level 1	Soil and soil extract
U-E-PFNS/K-PFOS	C8 H F15 O4 S	476.9273	Negative	Level 2&3	Soil extract
U-E-PFTriDS/K-PFDoDS	C12 H F23 O4 S	676.9138	Negative	Level 2&3	Soil extract
U-PFDoDS	C12 H F23 O3 S	660.9193	Negative	Level 2&3	Soil extract
U-PFDS	C10 H F19 O3 S	560.9263	Negative	Level 2&3	Soil extract

U-PFHpS	C7 H F13 O3 S	410.936	Negative	Level 2&3	Soil extract
U-PFNS	C9 H F17 O3 S	510.9294	Negative	Level 2&3	Soil extract
U-PFOS	C8 H F15 O3 S	460.9327	Negative	Level 2&3	Soil extract
U-PFTeDS	C14 H F27 O3 S	760.9139	Negative	Level 2&3	Soil extract
U-PFTriDS	C13 H F25 O3 S	710.9163	Negative	Level 2&3	Soil extract
U-PFUnDS	C11 H F21 O3 S	610.9242	Negative	Level 2&3	Soil extract

ESM D Results of AFFF standard oxidation

Table S5: Initial concentration of individual precursors, their terminal transformation products after the PhotoTOP, and mass balance (MB). Values marked as n.d. indicate that the compound was not detected.

**Precursor**	**Initial concentration of precursor (nM)**	**Transformation products (nM)**					**MB (%)**
		**PFBA**	**PFPeA**	**PFHxA**	**PFHpA**	**PFOA**	
PFHxSAm	26.0	1.541	1.0	21.7	n.d.	n.d.	90.1
PFOSAm	16.1	n.d.	n.d.	n.d.	0.3	14.9	94.7
PFHxSAm-Pr-DiMeAm	13.8	n.d.	0.4	16.3	n.d.	n.d.	120.5
6:2 FTSAm-Pr-B	15.5	n.d.	1.3	8.4	4.8	n.d.	93.4
6:2 FTSAm-Pr-DiMeNO	11.5	n.d.	1.9	11.6	5.1	n.d.	161.2
5:3 FTB	18.3	2.1	11.2	3.1	n.d.	n.d.	90.0
5:1:2 FTB	17.7	2.4	6.3	6.5	n.d.	n.d.	85.3

ESM E Results after direct soil and soil extract oxidation

Table S6: Molar concentrations (pmol/g) of precursors and transformation products (TPs) after PhotoTOP with 46 h oxidation of soil (C_{soil}) and soil extract ($C_{soil\ extract}$).

Name	Perfluorinated chain	C_{soil} (pmol/g)	$C_{soil\ extract}$ (pmol/g)
PFBA	3	30701	494
PFPeA	4	3474	1105
PFHxA	5	6098	2702
PFHpA	6	4439	1076
PFOA	7	4394	884
PFNA	8	1093	313
PFDA	9	359	63
PFUnDA	10	n.d.	5
PFDoDA	11	n.d.	1
PFBS	4	n.d.	7
PFPeS	5	n.d.	13
PFHxS	6	314	271
PFHpS	7	123	25.9
PFOS	8	2373	2478
PFNS	9	n.d.	8
PFDS	10	n.d.	2
PFDoDS	12	n.d.	1
PFHxSAm	6	297	n.d.
PFOSAm	8	72	1
6:2 FTCA	6	521	n.d.
6:2 FTSA	6	487	n.d.
5:1:2 FTB	5	386	1
5:3 FTB	5	77	n.d.
6:2 FTSAm-Pr-DiMeNO	6	n.d.	1

ESM F Results from qNTS study

Table S7: PFAS accounting for 90% of the total contamination identified in the qNTS approach [1].

Name	Concentration (µg/g)
6:2 FTSAm-Pr-B	3.17
6:2 FTSO-(2')OHPr-TriMeAm	2.29
6:2 FTSy-(2')OHPr-TriMeAm	1.88
PFOS	1.70
5:1:2 FTB	1.11
7:1:2 FTB	0.88
9:1:2 FTB	0.64
6:2 FTSAm	0.57
6:2 FTSA	0.38
8:2 FTSA	0.30
6:2 FTSAm-Pr-DiMeAm	0.20
7:3 FTB	0.19
5:3 FTB	0.13
9:3 FTB	0.13
PFHxS	0.13

ESM G Fate of PFAS classes in PhotoTOP

Table S8: Fate of PFAS classes in PhotoTOP. Note that the classification has limitations as some intermediates may also be precursors, or vice versa.

Class	Type
Cl-PFSA	Precursor
K-n:2 FTSA	Precursor
n:1:2 FTB	Precursor
n:2 FTB	Precursor
n:2 FTSAm-Pr-B	Precursor
n:2 FTSAm-Pr-DiMeAm	Precursor
n:2 FTSAm-Pr-DiMeNO	Precursor
n:2 FTSO-(2')OHPr-TriMeAm	Precursor
n:2 FTSO-Pr-Ad-(5',5')DiMeEtSA	Precursor
n:2 FTSy-(2')OHPr-TriMeAm	Precursor
n:2 FTSy-Pr-Ad-(5',5')DiMeEtSA	Precursor
n:2 FTTh-(2')OHPr-TriMeAm	Precursor
n:3 FTB	Precursor
n:4 FTB	Precursor
PFASAm-*N*-Me-*N*-EtA	Precursor
PFASAm-*N*-PrSA-*N*-Pr-DiMeAm	Precursor
PFASAm-Pr-B	Precursor
PFASAm-PrSA	Precursor
SF_5-PFSA	Precursor
SF_5-U-PFSA	Precursor
n:1:3 FTB	Intermediate
n:2 FTCA	Intermediate
n:2 FTSA	Intermediate
n:2 FTSAm	Intermediate
n:2 FTSAm-PrA	Intermediate
n:2 FTSy-PrA	Intermediate
n:2/m:2 FTSAm dimer	Intermediate
n:3 FTCA	Intermediate
PFASAm	Intermediate
PFASAm-Pr-DiMeAm	Intermediate
PFASAm-Pr-TriMeAm	Intermediate
PFASyA	Intermediate
U-n:2 FTSA	Intermediate
U-PFSA	Intermediate
PFCA	TP
U-E-PFSA/K-PFSA	Unclear
PFSA	Stable

ESM H Kinetics

Table S9: Fitting results with name of individual PFAS, oxidized matrix, applied model, initial concentration C_{start}, maximum concentration C_{max}, time when the formation rate is at its maximum t_{max}, formation rate k_f, decay rate k_d, and normalized root mean square error (RMSE). *C_{start} and C_{max} are expressed in µg/g for level 1 PFAS or as normalized response for level 2 and 3 PFAS.

Name	Oxidized matrix	Fitted model	C_{start} (*)	C_{max} (*)	t_{max} (h)	k_f (h^{-1})	k_d (h^{-1})	Normalized RMSE (%)
PFBA	Soil extract	Logistic formation	0.01	0.10	14.50	0.21		3.42
PFBA	Soil	Logistic formation	0.00	0.69	15.24	0.08		5.08
PFPeA	Soil extract	Logistic formation	0.03	0.27	13.04	0.31		3.28
PFPeA	Soil	Logistic formation	0.00	1.01	20.78	0.08		3.86
PFHxA	Soil extract	Logistic formation	0.01	0.81	12.79	0.36		2.33
PFHxA	Soil	Logistic formation	0.00	1.92	18.59	0.11		4.37
PFHpA	Soil extract	Logistic formation	0.00	0.38	10.72	0.33		3.20
PFHpA	Soil	Logistic formation	0.00	1.50	13.37	0.14		6.32
PFOA	Soil extract	Logistic formation	0.03	0.34	9.40	0.27		5.00
PFOA	Soil	Logistic formation	0.00	1.72	6.73	0.25		5.22
PFNA	Soil extract	Logistic formation	0.01	0.14	9.93	0.26		5.23
PFNA	Soil	Logistic formation	0.00	0.53	4.33	0.54		6.64
PFDA	Soil extract	Logistic formation	0.00	0.03	9.48	0.21		3.46
PFDA	Soil	Logistic formation	0.01	0.20	1.77	1.06		6.10
PFUnDA	Soil extract	Logistic formation	0.00	0.00	2.40	0.64		13.48
PFDoDA	Soil extract	Logistic formation	0.00	0.00	32.25	2.49		21.15
Cl-PFOS	Soil extract	Two-step exponential decay	0.83	0.56		0.63	0.14	4.74
SF5-PFHpS	Soil extract	Exponential decay	1.05				0.39	4.18
SF5-PFOS	Soil extract	Exponential decay	1.06				0.64	4.30
SF5-PFOS	Soil	Exponential decay	1.01				0.04	3.88
SF5-PFNS	Soil extract	Exponential decay	1.00				2.45	0.11
SF5-PFNS	Soil	Exponential decay	0.95				0.11	6.99

SF5-U-PFOS	Soil extract	Exponential decay	0.98				0.96	2.26
SF5-U-PFNS	Soil extract	Exponential decay	0.99				0.44	1.84
U-PFHpS	Soil extract	Logistic formation with exponential decay	1.61	1.33	23.20	0.15	0.02	4.50
U-PFOS	Soil extract	Two-step exponential decay	0.68	0.80		1.63	0.57	2.45
U-PFNS	Soil extract	Two-step exponential decay	0.79	0.92		1.13	0.70	2.76
U-PFDS	Soil extract	Two-step exponential decay	0.68	0.81		1.20	0.41	5.16
U-PFUnDS	Soil extract	Two-step exponential decay	0.65	0.80		1.20	0.34	3.93
U-PFDoDS	Soil extract	Two-step exponential decay	0.63	0.68		1.05	0.17	4.42
U-PFTriDS	Soil extract	Two-step exponential decay	0.66	0.47		0.78	0.08	5.64
U-PFTeDS	Soil extract	Two-step exponential decay	0.66	0.41		0.51	0.07	9.19
PFBSAm	Soil extract	Logistic formation with exponential decay	1015.47	1019.52	20.86	0.67	0.66	2.93
PFPeSAm	Soil extract	Logistic formation with exponential decay	625.84	625.08	20.57	0.63	0.62	3.90
PFPeSAm	Soil	Logistic formation with exponential decay	3.95	0.23	1.90	0.26	0.00	5.64
PFHxSAm	Soil extract	Logistic formation with exponential decay	23.43	20.33	19.81	0.51	0.49	4.53
PFHxSAm	Soil	Logistic formation with exponential decay	0.55	0.21	0.00	0.57	0.00	4.65
PFHpSAm	Soil extract	Logistic formation with exponential decay	85.30	69.67	19.83	0.48	0.42	6.35
PFOSAm	Soil extract	Logistic formation with	0.41	0.41	9.71	0.26	0.12	9.11

		exponential decay						
PFOSAm	Soil	Logistic formation with exponential decay	0.85	0.19	0.65	1.18	0.03	7.47
PFOSyA	Soil extract	Logistic formation with exponential decay	Overshooting		19.95	1.44	1.45	7.33
PFHxSAm-PrSA	Soil extract	Exponential decay	1.05				0.23	3.79
PFOSAm-Pr-B	Soil extract	Exponential decay	1.00				2.12	1.36
PFOSAm-Pr-B	Soil	Exponential decay	1.00				3.78	0.94
PFHxSAm-Pr-DiMeAm	Soil extract	Logistic formation with exponential decay	0.03	0.03	10.99	0.07	4.75	2.87
PFOSAm-Pr-DiMeAm	Soil extract	Logistic formation with exponential decay	0.77	2.38	11.58	0.00	1.00	11.23
PFHxSAm-Pr-TriMeAm	Soil extract	Logistic formation with exponential decay	4.21	0.42	11.62	0.00	0.67	9.10
PFOSAm-Pr-TriMeAm	Soil extract	Logistic formation with exponential decay	0.87	1.75	11.26	0.00	0.25	11.26
PFOSAm-Pr-TriMeAm	Soil	Logistic formation with exponential decay	1.20	1.64	11.17	0.00	1.23	2.52
PFHxSAm-N-PrSA-N-Pr-DiMeAm	Soil extract	Exponential decay	0.98				0.26	3.41
6:2 FTCA	Soil extract	Logistic formation with exponential decay	25.16	25.03	12.39	0.96	0.48	0.27
6:2 FTCA	Soil	Logistic formation with exponential decay	1.90	0.71	23.95	0.12	0.04	14.69
8:2 FTCA	Soil extract	Logistic formation with exponential decay	2.93	0.17	7.08	0.91	0.25	2.80
8:2 FTCA	Soil	Logistic formation with exponential decay	0.84	1.30	3.10	0.94	0.19	8.46

5:3 FTCA	Soil extract	Logistic formation with exponential decay	0.49	0.16	4.51	0.53	0.24	1.96
7:3 FTCA	Soil extract	Logistic formation with exponential decay	20.92	20.92	10.80	1.18	1.24	14.53
U-6:2 FTCA	Soil extract	Logistic formation with exponential decay	6.37	6.36	12.68	0.90	0.52	0.73
U-8:2 FTCA	Soil extract	Logistic formation with exponential decay	0.56	0.07	6.73	0.80	0.32	6.17
U-8:2 FTCA	Soil	Logistic formation with exponential decay	4.97	4.82	5.89	1.35	0.98	8.83
6:2 FTSA	Soil extract	Logistic formation with exponential decay	71.35	73.17	12.56	0.78	0.76	7.17
6:2 FTSA	Soil	Logistic formation with exponential decay	1.05	3.97	7.98	0.21	0.05	7.85
8:2 FTSA	Soil extract	Logistic formation with exponential decay	2.31	5.23	5.70	0.55	0.45	6.74
8:2 FTSA	Soil	Logistic formation with exponential decay	1.62	0.81	0.40	3.23	0.13	8.66
10:2 FTSA	Soil extract	Logistic formation with exponential decay	8.19	0.35	0.98	1.49	0.36	2.17
12:2 FTSA	Soil extract	Logistic formation with exponential decay	Overshooting		5.96	3.99	3.92	9.17
K-6:2 FTSA	Soil extract	Logistic formation with exponential decay	23.36	22.88	13.58	0.47	0.47	4.00
K-6:2 FTSA	Soil	Logistic formation with exponential decay	1.08	4.38	18.99	0.08	0.05	4.18
K-8:2 FTSA	Soil extract	Logistic formation with	1.87	7.25	6.10	0.44	0.34	4.89

		exponential decay						
5:1:2 FTB	Soil extract	Logistic formation with exponential decay	Overshooting		10.85	2.38	2.38	6.19
5:1:2 FTB	Soil	Logistic formation with exponential decay	12.78	13.43	33.42	0.14	0.14	4.24
7:1:2 FTB	Soil extract	Logistic formation with exponential decay	0.65	8.26	2.41	0.67	0.45	2.59
7:1:2 FTB	Soil	Logistic formation with exponential decay	1.91	1.07	0.00	0.30	0.25	1.71
9:1:2 FTB	Soil extract	Logistic formation with exponential decay	2.08	0.85	11.28	0.00	0.35	6.21
9:1:2 FTB	Soil	Logistic formation with exponential decay	222.56	221.54	4.41	2.45	3.10	0.25
11:1:2 FTB	Soil extract	Logistic formation with exponential decay	1.12	1.33	11.35	0.00	0.24	11.88
11:1:2 FTB	Soil	Logistic formation with exponential decay	0.66	2.90	11.50	0.00	0.68	3.78
5:3 FTB	Soil extract	Logistic formation with exponential decay	0.69	0.28	6.35	0.44	0.37	2.91
5:3 FTB	Soil	Logistic formation with exponential decay	2.22	2.05	27.57	0.11	0.11	1.58
7:3 FTB	Soil extract	Logistic formation with exponential decay	6.96	7.16	5.25	0.75	0.78	2.47
7:3 FTB	Soil	Exponential decay	1.01				0.18	1.88
9:3 FTB	Soil extract	Logistic formation with exponential decay	0.69	2.57	11.27	0.00	0.46	6.33
9:3 FTB	Soil	Logistic formation with	1.80	1.11	0.00	1.51	1.20	0.92

		exponential decay						
11:3 FTB	Soil extract	Logistic formation with exponential decay	0.71	2.24	11.61	0.00	0.46	11.20
5:1:3 FTB	Soil extract	Logistic formation with exponential decay	1.79	0.72	0.40	7.47	0.18	5.19
7:1:3 FTB	Soil extract	Logistic formation with exponential decay	1.00	1.30	0.42	10.00	0.22	4.35
6:2 FTB	Soil extract	Logistic formation with exponential decay	2.31	4.61	5.36	0.45	0.33	2.38
6:2 FTB	Soil	Logistic formation with exponential decay	302.66	296.57	28.89	0.39	0.40	3.30
8:2 FTB	Soil extract	Logistic formation with exponential decay	1.27	2.55	1.25	0.87	0.39	3.54
8:2 FTB	Soil	Exponential decay	0.99				0.38	4.12
6:4 FTB	Soil extract	Exponential decay	1.02				0.27	3.53
6:4 FTB	Soil	Exponential decay	0.96				0.40	4.59
6:2 FTSAm	Soil extract	Logistic formation with exponential decay	6.82	0.25	0.55	2.08	0.17	8.09
6:2 FTSAm-Pr-B	Soil extract	Exponential decay	0.15				0.64	3.84
6:2 FTSAm-Pr-B	Soil	Exponential decay	4.90				0.92	4.57
6:2 FTSAm-Pr-DiMeNO	Soil extract	Exponential decay	0.01				0.10	11.55
8:2 FTSAm-Pr-DiMeAm	Soil extract	Exponential decay	0.99				2.29	7.78
8:2 FTSAm-Pr-DiMeAm	Soil	Exponential decay	0.99				1.41	1.85
6:2 FTSAm-PrA	Soil extract	Logistic formation with exponential decay	1.46	0.96	0.20	3.86	0.22	4.29
6:2/6:2 FTSAm dimer	Soil extract	Logistic formation with exponential decay	6.91	0.55	2.86	0.64	0.25	10.77

6:2/6:2 FTSAm dimer	Soil	Logistic formation with exponential decay	1612.78	1612.77	2.22	6.67	7.37	0.47
6:2/8:2 FTSAm dimer	Soil extract	Logistic formation with exponential decay	1.84	1.46	3.10	0.45	0.13	12.71
6:2 FTTh-(2')OHPr-TriMeAm	Soil extract	Exponential decay	1.04				0.18	7.66
6:2 FTTh-(2')OHPr-TriMeAm	Soil	Exponential decay	0.98				0.16	3.43
6:2 FTSO-(2')OHPr-TriMeAm	Soil extract	Exponential decay	1.00				4.42	0.37
6:2 FTSO-(2')OHPr-TriMeAm	Soil	Exponential decay	0.99				2.19	3.08
6:2 FTSy-(2')OHPr-TriMeAm	Soil extract	Exponential decay	0.90				0.43	5.82
6:2 FTSy-(2')OHPr-TriMeAm	Soil	Exponential decay	0.98				0.26	1.23
8:2 FTSy-(2')OHPr-TriMeAm	Soil extract	Exponential decay	0.83				0.44	9.16
8:2 FTSy-(2')OHPr-TriMeAm	Soil	Exponential decay	1.06				0.38	6.40
6:2 FTSO-Pr-Ad-(5'5')DiMeEtSA	Soil extract	Exponential decay	1.00				3.16	1.39
6:2 FTSy-Pr-Ad-(5'5')DiMeEtSA	Soil extract	Exponential decay	1.06				0.23	4.00
8:2 FTSy-Pr-Ad-(5'5')DiMeEtSA	Soil extract	Exponential decay	1.07				0.25	4.70
10:2 FTSy-Pr-Ad-(5'5')DiMeEtSA	Soil extract	Exponential decay	0.95				0.36	9.95
6:2 FTSy-PrA	Soil extract	Logistic formation with exponential decay	1.53	1.32	0.65	1.42	0.21	4.51

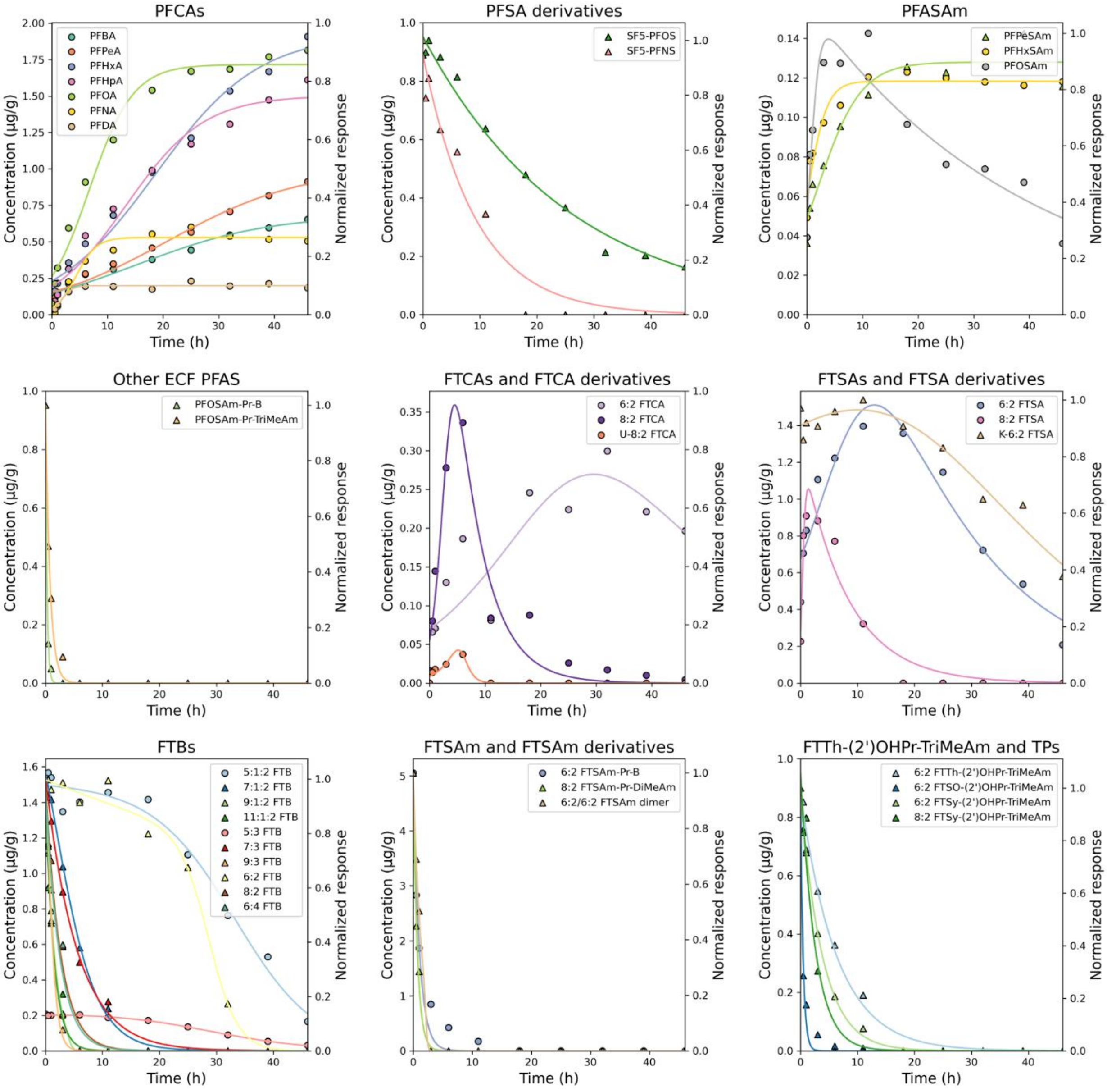

Figure S1: Fitted formation and degradation curves showing the concentration (µg/g) of level 1 PFAS (left axis, circles) and the normalized response of level 2 and 3 PFAS (right axis, triangles) during 46 h of direct soil irradiation.

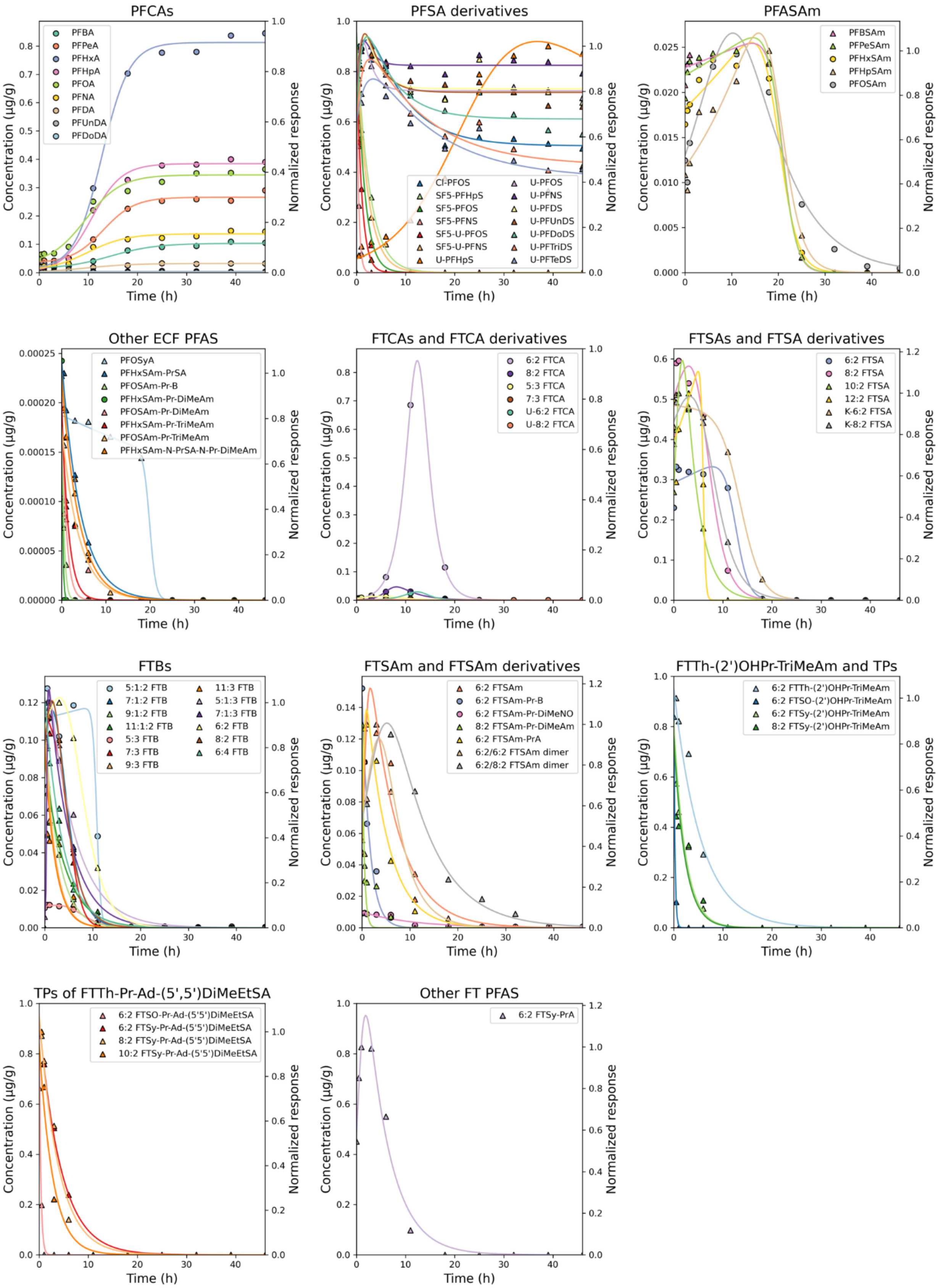

Figure S2: Fitted formation and degradation curves showing the concentration (μg/g) of level 1 PFAS (left axis, circles) and the normalized response of level 2 and 3 PFAS (right axis, triangles) during 46 h of soil extract irradiation.

ESM I Spearman correlations

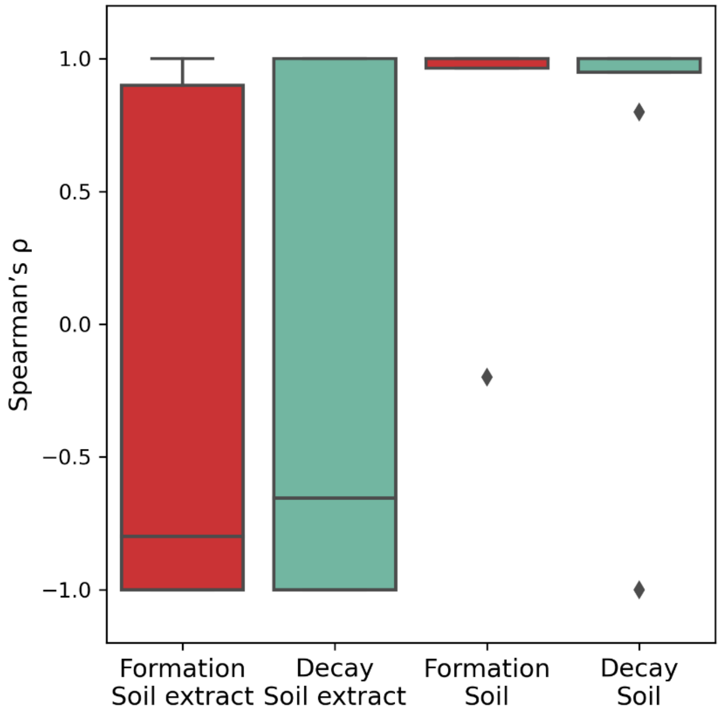

Figure S3: Boxplot of Spearman correlations between perfluorinated chain length and transformation or formation rates within individual PFAS classes.

References

1. Capitain C, Schüßler M, Bugsel B, Zweigle J, Vogel C, Leube P, Zwiener C (2025) Implementation of Matrix-Matched Semiquantification of PFAS in AFFF-Contaminated Soil. Environ Sci Technol 59 (14):7338-7347. doi:10.1021/acs.est.4c14255
